# Assessment of the prevalence and risk factors for dry eye symptoms among Romanian medical students using the ocular surface disease index – a cross-sectional study

**DOI:** 10.1186/s12886-023-03260-1

**Published:** 2024-01-05

**Authors:** Laura Denisa Preoteasa, Dana Preoteasa

**Affiliations:** 1 Carol Davila University of Medicine and Pharmacy, Dionisie Lupu street, no 37, Bucharest, 030167 Romania; 2 Department of Ophthalmology, Clinical Emergency Eye Hospital, Bucharest, Romania; 3 Onioptic Hospital, Craiova, Romania

**Keywords:** OSDI score, Ocular surface disease symptoms, Medical students, Risk factors, Prevalence

## Abstract

**Background:**

This study aims to assess how Romanian medical students suffer from dry eye disease symptoms, establish the prevalence and severity of dry eye (DE) symptoms and identify potential risk factors.

**Methods:**

An analytical, cross-sectional study was conducted on students from “Carol Davila” University of Medicine, Romania, after the final examination period of July 2022. The OSDI score (Ocular Surface Disease Index©) was applied in an online survey. The study adopted the standards used by other authors, who defined symptomatic DED as an OSDI score greater than 12. The chi-square test was used to establish statistical significance at a cutoff value of p < 0.05. The predictive model was created using linear logistic regression analysis. The goodness of fit of the logistic regression model was assessed using the Hosmer-Lemeshow test. When the severity outcome had a nominal categorical form, multinomial regression analysis with normal subjects as a reference was performed. The distribution of the severe type of symptomatology across sex categories and years of study was analyzed using a nonparametric test (Independent-Samples Kruskal-Wallis Test).

**Results:**

A total of 274 answers were received from 81.4% females and 18.6% males with a response rate of 35.58%. The mean age was 22.7 years ± 1.66 with an age range between 20 and 25 years old. Using the OSDI score, we established that the overall prevalence of DE symptoms was 83.6% (95%CI: 79.6%, 88%), with an 85.2% (95%CI: 80.5%, 89.8%) prevalence in females and 76.5% (95%CI: 65%,88%) in males. The severe form of DE was the most prevalent, regardless of the study year or sex. Increased screen time (*p*-value < 0.05) and non-smokers (*p*-value < 0.05) were proven risk factors. The predictive model which includes the explanatory variables (sex, contact lens wearers, smoking, oral contraceptives, screen time) proved an 84.7% predictability for symptomatic DE and was able to better predict the dependent variable than the intercept model only (*p*-value < 0.05). Smoking (*p* = 0.002) and screen time (*p* = 0.009) preserved their significance in the multinominal regression as well.

**Conclusions:**

This is the first study to report the epidemiology of DE symptoms among Romanian medical students. OSDI revealed a high prevalence of symptomatic DE in medical students. Screen time, although not the only factor, likely plays a role in exacerbating the disease. This information can be used to inform healthcare policies, establish occupational health guidelines, and implement preventive measures for individuals in similar high-stress academic or professional environments.

**Supplementary Information:**

The online version contains supplementary material available at 10.1186/s12886-023-03260-1.

## Introduction

Dry eye disease (DED) is a condition that affects one in five adults and can significantly lower quality of life due to irritation, discomfort, visual disturbances, and ocular fatigue [1]. The epidemiology of DED remains a challenging task due to the lack of correlation between signs and symptoms and high interindividual variability [2, 3]. However, DED worldwide is very common, which results in a considerable overall humanistic and economic burden, especially in young populations, due to decreased work productivity [4], difficulty regarding reading and driving [5] and impact on physical and social functioning [6].

Dry eye disease was first defined in 1995 by the National Eye Institute, which was later improved by the Definition and Classification Subcommittee of the International Dry Eye WorkShop in 2007 (DEWS) and 2017 (DEWS II) to: “Dry eye is a multifactorial disease of the ocular surface characterized by a loss of homeostasis of the tear film, and accompanied by ocular symptoms, in which tear film instability and hyperosmolarity, ocular surface inflammation and damage, and neurosensory abnormalities play etiological roles” [2]. The primary focus was on the role of inflammation in the pathogenesis of DED which led to loss of homeostasis and neurosensory implications.

Etiopathogenesis identifies two main causes: aqueous tear deficiency (divided into Sjogren and Non-Sjogren dry eye) and increased evaporation caused by intrinsic (Meibomian gland dysfunction, abnormal lid function, reduced blinking, drugs such as antihistamines, beta-blockers, spasmolytics, diuretics) or extrinsic factors (Hypovitaminosis A, topical drugs, contact lens wear, ocular surface disorder – allergic conjunctivitis) [7, 8, 9]. There are several hybrid types of DED, where lacrimal insufficiency and increased evaporative loss interact to develop ocular surface hyperosmolarity (e.g. Sjogren’s syndrome) [9]. Sjogren’s syndrome is an autoimmune disease caused by lymphocytic infiltration of lacrimal and salivary glands resulting in sicca symptoms [10], accompanied by serological features (anti-Ro and anti-La antibodies, rheumatoid factor, anti-tissue antibodies) [11]. Non-Sjogren DE (NSDE) is a term used to define a group of local and systemic disorders (lacrimal gland deficiency, constricted lacrimal gland duct, reflex hyposecretion) [7, 8] in the absence of systemic autoimmune disease [11].

The diagnosis of DED is not based on a single definitive investigation, but rather on a practical sequence of tests to evaluate the prevalence of symptoms, tear stability, ocular surface staining and reflex tear flow [3]. Quantifying ocular symptoms by means of questionnaires represents a crucial screening tool that can determine whether further tests are needed [12]. Multiple questionnaires have been developed to evaluate patients’ perception of DED [12]. The OSDI (Ocular Surface Disease Index) was proven to be a highly reliable test, effectively discriminating between normal, mild, moderate, and severe DED, which reduces survey bias [13]. In the objective diagnosis of DED, physicians use the results of OSDI score > = 13 plus at least one abnormal result of the markers of homeostasis (non-invasive tear breakup time, osmolarity, ocular surface staining with fluorescein or lissamine green) [12].

The present study aims to investigate the prevalence of dry eye symptoms among Romanian medical students, find possible correlations between year of study and degree of severity and establish the prevalence of known associated risk factors for our study population. Conducting a study on the prevalence of dry eye disease among medical students can have implications for individual health, academic performance, occupational health policies, and contribute to the broader understanding of eye health in high-stress academic settings. At present, there are no studies regarding the prevalence and severity of DED in Romania, let alone the young population.

Medical students often spend extended hours studying, using digital devices, and in clinical settings. Identifying a high prevalence of dry eye disease can highlight the occupational impact of their activities and planprompt actions to mitigate potential harm. Knowledge about the signs and symptoms of dry eye disease can lead to the development of targeted educational interventions such as workshops on proper eye care, ergonomic adjustments in study environments, and the importance of regular breaks to reduce eye strain.

By identifying and addressing the prevalence of dry eye symptoms, there is potential to improve the overall quality of life for medical students. This can positively impact their academic performance, general well-being, and long-term eye health. The study may contribute valuable data to the broader field of medical knowledge, shedding light on the prevalence of dry eye disease in a specific demographic and potentially informing future research directions into investigating the impact of specific study habits, stress levels, or environmental factors on eye health.

## Materials and methods

The principles of the Declaration of Helsinki were followed in this study. Ethical approval was received beforehand from the Ethics Committee of Onioptic Ophthalmology Hospital (730/01.07.2022).

The Strengthening the Reporting of Observational Studies in Epidemiology (STROBE) guidelines were followed in conducting and reporting this cross-sectional, analytical study [14]. An online survey (Google Form) was distributed among 770 students from “Carol Davila” University of Medicine and Pharmacy (UMFCD), Bucharest, Romania, after the final examination period in July 2022. A total of 274 students enrolled electively in the study with a 35.58% response rate. The inclusion criteria were represented by any medical student aged between 18 and 28 years old who was currently enrolled in any undergraduate program at UMFCD who provided informed consent for the study and understood Romanian language. Students who do not understood Romanian language, who had recent eye surgery or active ocular infections or inflammation and those who were diagnosed with other systemic diseases beside those mentioned in the questionnaire were excluded. A participant information sheet was attached at the beginning of the survey that underlined voluntary submission which was signed by all participants. The data were kept private.

The formula: *n* = [(Z)^2^p(1-p)] /δ^2^, where Z is the value based on a confidence level = 1.96 using a 95% CI, p is the sample proportion = 50% and δ is the margin of error = 5% was applied to determine appropriate sample size. Since DED among medical students from UMFCD had never been estimated before, an anticipated population percentage (P) of 50% was used, obtaining an ideal sample size equal to 385.

The Diagnostic Methodology Subcommittee considered dry eye as a “chronic, symptomatic, ocular surface disease, which may, however, occasionally be asymptomatic” [3]. Asymptomatic dry eye refers to the fact that even if subjectively the patient does not have any complaints during ophthalmological examination, objective criteria of dry eye can be discovered such as: tear hyperosmolarity, corneal fluorescein staining or decreased tear break up time. Classical symptomatic dry eye is based on both experiencing the symptoms of dry eye and exhibiting objective signs of aqueous tear deficiency or increased evaporation [3].

A self-administered Romanian questionnaire **(Supplementary material** 1.) was distributed among medical students which included background information (age, sex, study year), closed-ended questions about the presence or absence of several risk factors (the average daily screen time, smoking, use of contact lenses, atopy, oral contraceptives, autoimmune disease, vitamin A deficiency, isotretinoin treatment, antimuscarinic treatment, congenital cataract, keratoconus, and history of refractive surgery) and the approved Romanian translation of OSDI index (**Supplementary material **2). Some risk factors such as autoimmune disease, vitamin A deficiency, keratoconus and congenital cataract were valid only if the person has a diagnosis confirmed by a specialist or is undertaking medication prescribed by a physician, which was specified at the respective question. The authors have permission to use this instrument from the copyright holders © 1995 Allergan Inc. There were no open-answer questions, and all items were mandatory for the submission of the questionnaire to avoid missing data. The OSDI is a 12-item self-administered questionnaire, with each item being scored on a scale from 0 (none of the time) to 4 (all of the time) [15]. The final score is calculated using the formula: $OSDI = \frac{{\left( {SUM\,OF\,SCORES} \right) \times 25}}{{NUMBER\,OF\,QUESTIONS\,ANSWERED}}$. Based on this result, the patients can be divided into the following categories: no symptoms of DE (0–12), mild symptoms of DE (13–22), moderate symptoms of DE (23–32) and severe symptoms of DE (33–100) [15]. The study adopted the standards used by other authors, who defined symptomatic DED as an OSDI score greater than 12 [13, 16]. The OSDI score demonstrates both high internal consistency (the Cronbach α for the overall instrument and each of the subscales ranged from good to excellent) and good to excellent test-retest reliability in a large sample of patients with dry eye disease and normal controls [13].

Categorical nominal variables included sex, smoking, use of contact lenses, atopy, oral contraceptives, autoimmune disease, vitamin A deficiency, isotretinoin treatment, antimuscarinic treatment, congenital cataract, keratoconus, and history of refractive surgery. Categorical ordinal variables included study year (1st to 6th year) and the average daily screen time (hours): (1) 1–3 h/day, (2) 3–5 h/ day, (3) 5–8 h/ day, (4)  > 8 h/day. Continuous numeric variables were represented by OSDI scores obtained for each student. The outcomes included “yes” if OSDI > 12 and “no” if OSDI was less than or equal to 12 for the prevalence of DE symptoms. Severity outcomes were also based on the OSDI score: no symptoms of DE (0–12), mild symptoms of DE (13–22), moderate symptoms of DE (23–32) and severe symptoms of DE (33–100). The aim of the study was to classify dry eye among medical students according to the degree of severity and find possible correlations with susceptible contributing factors.

The statistical analysis was carried out using SPSS Statistics 26.0. Frequencies and percentages were employed for the descriptive part of the study. The Odds Ratio was calculated using SPSS statistical software. The chi-square test was used to establish statistical significance at a cut-off value of p < 0.05. Ordinal variables were compared using nonparametric tests such as the Mann-Whitney U test. The predictive model was created using linear logistic regression analysis. The model included predictors that add statistical value. The goodness of fit of the logistic regression model was evaluated using the Hosmer and Lemeshow Statistic. Similarly, when the severity outcome had a nominal categorical form, multinomial regression analysis with normal subjects as a reference was performed.

## Results

A total of 274 answers were obtained, of which 223 (81.4%) were female and 51 (18.6%) were male. The Mean age was 22.7 years ± 1.66 with an age range between 20 and 25 years old. The mean age of males was 22.56 ± 1.7 years, and the mean age of females was 22.72 ± 1.65 years. Population distribution by year of study and gender is found in Table 1.

**Table 1:** Sample distribution by year of study and gender

	No. of students	% of students	Females	Males
First year	29	10.6%	22 (75.86%)	7 (24.13%)
Second year	46	16.8%	38 (82.60%)	8 (17.40%)
Third year	60	21.9%	47 (78.33%)	13 (21.66%)
Fourth year	43	15.7%	37 (86.04%)	6 (13.95%)
Fifth year	38	13.9%	32 (84.21%)	6 (15.79%)
Sixth year	58	21.2%	47 (81.03%)	11 (18.96%)

Using the OSDI score, we established that the overall prevalence of DE symptoms was 83.6% (95% CI: 79.6%, 88%), with an 85.2% (95%CI: 80.5%, 89.8%) prevalence in females and 76.5% (95% CI: 65%,88%) in males. The mean OSDI score for males was 25.73 (95%CI: 20.91, 30.55) and for females was 31.17 (95%CI: 28.75, 33.59).

**Table 2:** Distribution of DE severity among sample population

	No. of students	% of students	95% CI
No symptoms of DE	45	16.40%	12%, 20.8%
Mild DE symptoms	61	22.20%	17.3%, 27.2%
Moderate DE symptoms	52	19.00%	14.3%, 23.6%
Severe DE symptoms	116	42.33%	36.5%, 48.2%

**Table 3:** Distribution of DE severity according to sex

	Female	95% CI	Male	95% CI
No symptoms of DE	14.80%	10.10%, 19.40%	23.53%	11.90%, 35.17%
Mild DE symptoms	21.52%	16.13%, 26.9%	25.50%	13.53%, 37.45%
Moderate DE symptoms	20.18%	14.9%, 25.45%	13.70%	4.28%, 23.17%
Severe DE symptoms	43.50%	37.00%, 50.00%	37.25%	24.00%, 50.52%

43.5% (95% CI: 37.00%, 50.00%) of females and 37.25% (95% CI: 24.00%, 50.50%) of men experienced severe symptomatology. Similarly, there were more males (23.53%) versus females (14.8%) without symptoms of dry eye -Table 3.

The Kolmogorov test indicate that the distribution of OSDI score in males is taken from a normal population (*p* = 0.180), while in females the *p* value = 0.003 does not follow normal distribution. The distribution of OSDI score and DE severity regarding the severe type of symptomatology was found to be consistent across sex categories after performing a nonparametric test (Independent-Samples Kruskal-Wallis Test).

The overall prevalence of severe DE symptoms was 42.33% (95% CI: 36.5%, 48.2%) – Table 2. The severe form of DE was the most prevalent, regardless of the year of study (Table 4). The same nonparametric test (Independent-Samples Kruskal-Wallis Test) confirmed that there was no significant difference regarding the severe form among study year.

**Table 4:** Distribution of DED severity according to study year

	1st year	2nd year	3rd year	4th year	5th year	6th year
No symptoms of DED	24.1%	10.9%	11.7%	16.3%	21.1%	16.4%
Mild DED symptoms	13.8%	37.0%	21.7%	20.9%	18.4%	22.3%
Moderate DED symptoms	10.3%	15.2%	26.7%	18.6%	23.7%	19.0%
Severe DED symptoms	51.7%	37.0%	40.0%	44.2%	36.8%	42.3%

**Fig. 1:**
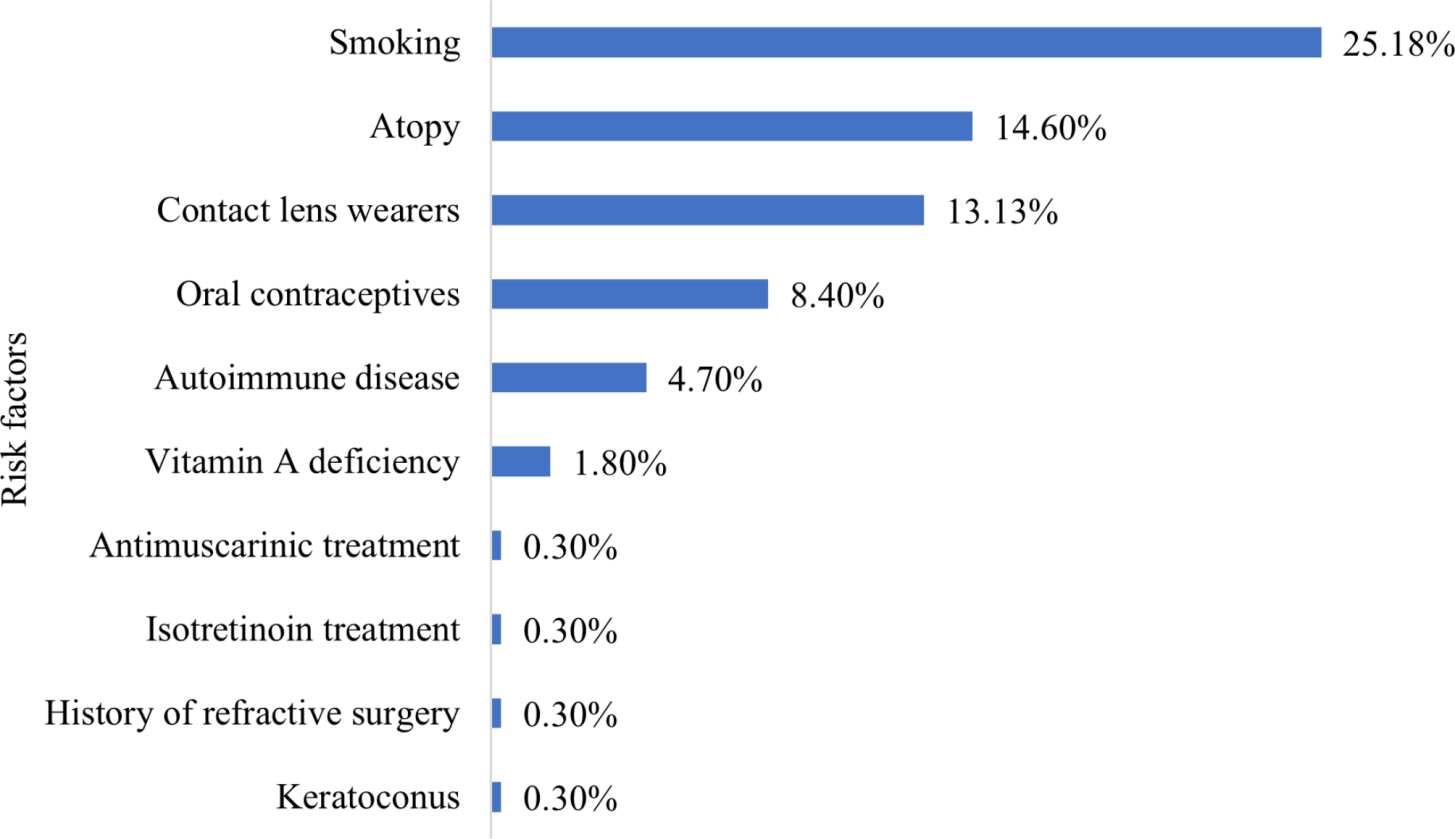
Frequency of risk factors among sample population

While 39.4% of students do not encounter any risk factor, the other 60.6% can be divided into multiple risk profiles (Fig. 1).

The prevalence of DE among possible risk factors encountered and the statistical significance (*p*-value) of every risk factor causing DE is detailed in Table 5.

**Table 5:** Prevalence of DED among possible risk factors

		DED		*P* value
		**NO**	**YES**	
Sex	Male	23.5% (12)	76.5% (39)	.
	Female	14.8% (33)	85.2% (190)	0.129
Screen time	1–3 h/ day	30.8% (4)	69.2% (9)	.
	3–5 h/ day	22.9% (16)	77.1% (54)	0.092
	5–8 h/day	14.4% (17)	85.6% (101)	0.433
	> 8 h/day	11.0% (8)	89.0% (65)	0.141
Study year	1st year	24.1% (7)	75.9% (22)	.
	2nd year	10.9% (5)	89.1% (41)	0.265
	3rd year	11.7% (7)	88.3% (53)	0.260
	4th year	16.3% (7)	83.7% (36)	0.978
	5th year	21.1% (8)	78.9% (30)	0.407
	6th year	19.0% (11)	81.0% (47)	0.556
Risk factors	Yes	18% (27)	82.0% (123)	.
	No	14.5% (18)	85.5% (106)	0.438
Smoking	Yes	27.5% (19)	72.5% (50)	0.004
	No	12.7% (26)	87.3% (179)	.
Atopy	Yes	10.0% (4)	90% (36)	0.235
	No	17.5% (41)	82.5% (193)	
Contact lens wearers	Yes	8.3% (3)	91.7% (33)	0.160
	No	17.6% (42)	82.4% (196)	.
Oral contraceptives	Yes	8.7% (2)	91.3% (21)	0.296
	No	17.1% (43)	82.9% (208)	.
Autoimmune disease	Yes	15.4% (2)	84.6% (11)	0.918
	No	16.5% (43)	83.5% (218)	.
Vitamin A deficiency	Yes	0% (0)	100% (5)	0.319
	No	16.7% (45)	83.3% (224)	.
Isotretinoin treatment	Yes	0% (0)	100% (1)	0.657
	No	16.5% (45)	83.5% (228)	.
Congenital cataract	Yes	0% (0)	100% (1)	0.657
	No	16.5% (45)	83.5% (228)	.
Antimuscarinic treatment	Yes	0% (0)	100% (1)	.
	No	16.5% (45)	83.5% (228)	0.657
History of refractive surgery	Yes	0% (0)	100% (1)	0.657
	No	16.5% (45)	83.5% (228)	.
Keratoconus	Yes	0% (0)	100% (1)	0.657
	No	16.5% (45)	83.5% (228)	.

On average, a medical student regardless of study year spends between 5 and 8 h/day (43.07%, 95% CI: 37.20%, 48.93%) in front of blue screens. Smoking was encountered in 25.18% of the population, with a prevalence of DE of 18.24% (95% CI: 13.67%, 22.82%) of cases. It was followed by atopy (14.6%), with a prevalence of DE of 13.14% (95% CI: 9.14%, 17.14%). Contact lens wearers were identified in 13.13%, with a prevalence of DE of 12.04% (95% CI: 8.19%, 15.9%). Use of oral contraceptives was reported in 8.4% of cases, leading to DE in 7.66% (95% CI: 4.51%, 10.81%) of cases. Autoimmune diseases were determined in 4.7%, with a prevalence of DE of 4.01% (95% CI: 1.69%, 6.34%) and vitamin A deficiency was found in 1.82%, leading to DE in 1.8% (95% CI: 0.24%, 3.41%) of cases. Other risk factors such as keratoconus, history of refractive surgery, isotretinoin treatment, antimuscarinic treatment and history of congenital cataract had a low frequency among the target population (0.3%). The only proven statistical association was between nonsmokers and dry eye symptoms (*p*-value < 0.05).

For each risk factor, the odds ratio (OR) and 95% CI for developing DE were calculated using SPSS (Table 6). Nonsmokers proved a higher risk of developing symptoms of DE than smokers (OR = 1.353, 95% CI: 1.044, 1.753) with statistical significance.

**Table 6:** Independent Odds ratio for developing DED among study group

	OR for DED	95% CI for RR	*P* value
Sex (woman)	1.131	0.940, 1.362	0.098
Screen time			
1–3 h/day	1.0		
3–5 h/day	1.5	0.407, 5.522	0.542
5–8 h/day	2.641	0.731, 9.545	0.139
> 8 h/day	3.611	0.901, 14.469	0.070
Smoking (no)	1.353	1.044, 1.753	0.005
Atopy (yes)	1.769	0.662, 4.723	0.170
Contact lens wearers (yes)	0.917	0.834, 1.008	0.118
Oral contraceptives (yes)	2.063	0.501, 8.491	0.235
Autoimmune disease (yes)	1.081	0.248, 4.712	0.638

Logistic regression analysis was performed to explain the relationship between DE and sex, contact lens wearers, smoking, oral contraceptives and screen time (Table 7). Both Kendall’s tau-b and Spearman coefficient indicate a correlation between Atopy and Oral contraceptives, therefore Atopy was removed from our model. The null model correctly predicts 83.6% of cases with DE (*p*-value < 0.05). The novel model which includes the explanatory variables increases its predictability to 84.7%. It was evaluated with the Hosmer and Lemeshow goodness-of-fit test: *p*-value = 0.672 and chi-square = 4.903. The Omnibus test concluded that the new model with the explanatory variables included is an improvement over the baseline model (*p*-value < 0.05, chi-square = 20.313).

**Table 7:** Results of logistic regression analysis

	Regression coefficient	Wald statistic	*P* value	Odds Ratio	95% CI for OR
Sex (woman)	0.531	1.732	0.188	1.70	0.77, 3.752
Contact lens wearers (yes)	0.797	1.499	0.221	2.219	0.619, 7.952
Smoking (no)	1.138	9.902	0.002	3.12	1.536, 6.338
Oral contraceptives (yes)	0.657	0.714	0.398	1.930	0.42, 8.868
Screen time		7.487	0.058		
1–3 h/day					
3–5 h/day	0.466	0.460	0.498	1.594	0.414, 6.130
5–8 h/day	1.041	2.306	0.129	2.832	0.739, 10.85
> 8 h/day	1.601	4.686	0.030	4.960	1.163, 21.142
Overall Statistics				0.005	

In the multinomial logistic regression, DE severity was compared, using the normal category as reference (Table 8**).** The full model statistically significantly predicts the dependent variable better than the intercept model only (*p*-value < 0.05). The model fits the data well according to goodness-of-fit (*p*-value = 0.425, Pearson chi-square = 70.564). Smoking preserves its significance in the multinomial regression as well. There was a significant difference between the normal form and the severe form of DE. The number of hours spent in front of blue screens proved to be significant for developing the severe symptoms of DE: students who spent over 8 h/day were compared to those who spent 3–5 h/day (*p* < 0.05) and 1–3 h/day (*p* < 0.05) (Table 8).

**Table 8:** Results of multinomial logistic regression analysis

DED severity		Regression coefficient	Wald	*P*-value	OR	95% CI for OR	
**Mild**	Intercept	,248	,033	,856			
	[Sex = female]	,364	,584	,445	1,439	,566	3,662
	[Sex = male]	0	.	.	.	.	.
	**[Smoking = no]**	,**921**	**4,421**	,**035**	**2,511**	**1,064**	**5,922**
	[Smoking = yes]	0	.	.	.	.	.
	[Screen = 1–3 h/ day]	-,840	1,045	,307	,432	,086	2,161
	[Screen = 3–5 h/ day]	-,845	2,220	,136	,430	,141	1,305
	[Screen = 5–8 h/ day]	-,682	1,525	,217	,505	,171	1,493
	[Screen = > 8 h/ day]	0	.	.	.	.	.
	[Contact lens wearers = no]	-,268	,119	,730	,765	,167	3,509
	[Contact lens wearers = yes]	0	.	.	.	.	.
	[Oral contraceptives = no]	,007	,000	,994	1,007	,157	6,466
	[Oral contraceptives = yes]	0	.	.	.	.	.
**Moderate**	Intercept	,796	,351	,554			
	[Sex = female]	,786	2,039	,153	2,194	,746	6,450
	[Sex = male]	0	.	.	.	.	.
	**[Smoking = no]**	**1,179**	**6,156**	,**013**	**3,250**	**1,281**	**8,246**
	[Smoking = yes]	0	.	.	.	.	.
	[Screen = 1–3 h/ day]	-1,457	2,096	,148	,233	,032	1,674
	[Screen = 3–5 h/ day]	-1,130	3,324	,068	,323	,096	1,088
	[Screen = 5–8 h/ day]	-,371	,425	,515	,690	,226	2,107
	[Screen = > 8 h/ day]	0	.	.	.	.	.
	[Contact lens wearers = no]	-1,207	2,836	,092	,299	,073	1,219
	[Contact lens wearers = yes]	0	.	.	.	.	.
	[Oral contraceptives = no]	-,494	,292	,589	,610	,102	3,660
	[Oral contraceptives = yes]	0	.	.	.	.	.
**Severe**	Intercept	2,065	3,035	,081			
	[Sex = female]	,535	1,423	,233	1,707	,709	4,112
	[Sex = male]	0	.	.	.	.	.
	**[Smoking = no]**	**1,252**	**9,710**	,**002**	**3,498**	**1,591**	**7,689**
	[Smoking = yes]	0	.	.	.	.	.
	**[Screen = 1–3 h/ day]**	**-2,552**	**6,756**	,**009**	,**078**	,**011**	,**534**
	**[Screen = 3–5 h/ day]**	**-1,320**	**6,166**	,**013**	,**267**	,**094**	,**757**
	[Screen = 5–8 h/ day]	-,583	1,347	,246	,558	,209	1,494
	[Screen = > 8 h/ day]	0	.	.	.	.	.
	[Contact lens wearers = no]	-,816	1,435	,231	,442	,116	1,681
	[Contact lens wearers = yes]	0	.	.	.	.	.
	[Oral contraceptives = no]	-1,003	1,557	,212	,367	,076	1,773
	[Oral contraceptives = yes]	0	.	.	.	.	.

The bold lines represent the significant differences between the respective DED form (mild, moderate or severe) and the normal category used for reference.

## Discussion

While most studies evaluating the impact of DE are based on older populations and an increase in severity with older age, we would like to address the issue among youths, which proved of equally significant importance. The epidemiology of DE in Romania among medical students is reported in this paper for the first time.

In our study, a total of 274 answers were obtained, of which 223 (81.4%) were female and 51 (18.6%) were male. The ratio between women and men is similar to that published by Statista in 2023, stating that 89.5% of medical staff with upper secondary education in Romania are women [17].

The prevalence of ocular surface disease symptoms at the end of finals period in July 2022 was 83.6% (95% CI: 79.6%, 88%), with an 85.2% (95%CI: 80.5%, 89.8%) prevalence in females and 76.5% (95% CI: 65%,88%) in males. In the TFOS DEWS II Epidemiology Report, the prevalence of disease for studies involving symptoms with or without signs ranged from approximately 5–50% [2]. In a similar study conducted in Poland on university students, the prevalence of DE symptoms among the study population (45.8% medical students and 54.2% nonmedical students) was 57.1% [16]. Based on the same diagnostic criteria (OSDI score > 12) and using the student target population, cross-sectional surveys revealed that DE prevalence varied little between nations – 62.6% in Dubai [18], 60.5% in Serbia [19], 70.8% in Thailand [20] and 70.9% in Peru [21]. In Spain, research conducted on college students enrolled in e-learning courses used a cut-off value of OSDI > 22 to define symptomatic dry eye and obtained a prevalence equal to 51.8% [22].

One reason our study proved a higher prevalence may be the time period in which students were asked to complete the questionnaire, that is after intensive study period. Medical students require more hours to prepare for their exams associated with heavy psychological stress and lack of sleep during this period [23]. It is known that these factors increase ocular fatigue and predispose individuals to exacerbation of symptoms [24–27]. A study based on high GPA medical students revealed that most of them study approximately 3–4 h/day, in addition to going to classes, with more than 83% using lecturer slides and 76.1% using video software [28], all of which result in increased screen time and ocular fatigue. The following study’s results may differ from other studies in other ways, including the mean age of the participants, the online acquisition of data, climatic variations, free time habits and lifestyle discrepancies [29]. It should also be considered that the present study was carried out in the post pandemic period of COVID-19, when medical students take classes in an e-learning environment, while most literature studies provided were conducted before this period. Self-selection bias is a limitation of our study, as data was collected only from participants who enrolled themselves in the study and not from the whole population of students from UMFCD. Students who do not experience DE symptoms were presumed to be less likely to answer the questionnaire, resulting in a high rate of false positives. In this case, people who responded to our survey may not be truly a random sample. Besides, OSDI questionnaire was delivered through online means, thus increasing the possibility of overestimation.

Logistic regression modeling was conducted to understand the relationship between risk factors and DE among the study participants. The most frequently reported risk factor was smoking (25.18%). It demonstrated a statistically significant detrimental effect, increasing the likelihood of DE symptoms among nonsmokers compared to smokers. In this case, it is important to remember that the OSDI is a subjective questionnaire, and objective tests should be correlated to establish the effect of smoking on dry eye disease (DED). We consider that smoking was significant in a negative way due to its effect of lowering corneal sensitivity [30], thus producing fewer upsetting symptoms. The literature provides divided results regarding the influence of smoking on ocular surface symptoms. A systemic review and meta-analysis based on a total of 22 studies (4 cohort and 18 cross-sectional studies) including 160.217 subjects concluded that there is no significant relationship between current smokers (OR adjusted = 1.14; 95% CI: 0.95–1.36; *p* = 0.15; I2 = 84%) and former smokers (OR adjusted = 1.06; 95% CI: 0.93–1.20; *p* = 0.38; I2 = 26.7%) for the risk of DED [31]. Another analysis based on a large-scale multicenter randomized clinical trial of patients with moderate to severe DE found a significant association between daily smoking and DED (*p*-value = 0.047) [32]. Even in the TFOS DEWS II study, smoking was categorized as an inconclusive risk factor regarding DE [2].

Another risk factor established in our study was increased screen time. Students spending over 8 h/day in front of blue screen had a higher chance of developing DED. Screen time maintained its significance in the multinomial regression as well, concluding that students who spend less than 5 h/day using displays had a lower chance of developing severe DED. Numerous large cross-sectional studies have confirmed the high prevalence of dry eye symptoms among display users, especially in the young population [16, 18, 19, 33–36].

Female sex has been linked to a higher incidence of DE symptoms in multiple epidemiological studies [8, 22, 36, 37]. In our study, women do not prove a significant higher chance than men of developing symptoms of DED (OR = 1,131, 95% CI: 0.940, 1.362, *p* = 0.1). According to the Dry Eye Workshop II, only populations 50 years old and above show a statistically significant gender difference in symptomatic patients [38]. Other risk factors confirmed in other studies such as contact lenses [2, 16, 19, 22, 36, 39, 40], allergies [16, 19, 36] or oral contraceptives [8, 41, 42]were not statistically significant in our population. History of refractive surgery, which was a probable risk factor in TFOS DEWS II, had a very low incidence in our population to provide relevant information [2, 38]. In a cross-sectional study in Netherlands, independent risk factors associated with DED included female sex, contact lens use, keratoconus, allergic conjunctivitis, Bell’s palsy, Graves’ disease, glaucoma (treated with either drops or surgery), cataract surgery, refractive surgery, autoimmune disorders, liver cirrhosis, psychiatric pathologies, atopy, osteoporosis, sinusitis and sleep apnea [43]. Interestingly, ex-smokers showed higher rates of DED than non-smokers or active smokers [43]. High blood pressure and high BMI were strongly associated with less dry eye [43]. Contact lens use was a strong risk factor in younger age categories [43].

The severe form of DE was the most prevalent, regardless of the year of study. We want to emphasize that there was no difference regarding study year in terms of severity, concluding that the amount of visual effort does not increase in higher university years. Similarly, there was no significant difference between females and males regarding the severity of DE.

The whole population of medical students in Romania was not included in our study because it was geographically restricted to the University of Medicine and Pharmacy “Carol Davila”. This leads to sampling bias and lack of external validity, as the results cannot be generalized. Similar future studies should be performed in different medical universities in Romania to be able to compare the results.

Another element of bias is represented by the closed-ended questionnaire, which is useful for gathering data and standardizing responses, but the respondents have a limited number of choices and the information collected may be incomplete or inaccurate. Some risk factors such as quality of sleep [26, 27], medical history [16] or mental health [25] were not included. Furthermore, the OSDI score does not address tearing or foreign body sensation, making it less accurate in some symptoms that the patient may experience [44, 45]. In addition, OSDI highlights frequency rather than severity [44, 46]. Plus, it is a subjective test based solely on symptoms and how they are perceived by every individual. We did not use any objective tests to confirm the score. Moreover, the data were collected after the finals period, in which medical students suffer from increased visual stress, especially due to visual display terminals used for studying and long study hours needed for preparation. The questionnaire should be repeated on the same subjects at a different time period to be able to make a comparison between the prevalence of DE after intensive studying and the prevalence of DE in medical students in general.

Our study uses a cross-sectional research design, thus both the exposure and the results are evaluated at the same time. Therefore, the assessment of a temporal link between risk factors and the presence of a high OSDI score is limited. Thus, it is not feasible to establish a real cause-effect relationship.

The study followed a convenience sampling strategy. Possible bias includes population bias (medical students), geographical bias (subjects from UMFCD), subjective bias based only on dry eye symptoms and response bias. It is important to note that these results should be interpreted with caution because the sample size was slightly smaller than the optimal one and all data were self-reported and subjective. Unfortunately, the response rate among students could not be planed, as they should enroll themselves in this study. In addition, there was a large difference in the sample size between students with and without DE, which prevented us from having a control group of similar size. Taking this into account, we believe that the population under research is still accurately represented by this study.

## Conclusions

This is the first cross-sectional study to assess the prevalence of symptomatic DE among Romanian medical students. It revealed a high prevalence of self-reported ocular surface symptoms in medical students (83.6%), with the severe form being the most common, regardless of study year (42.3%). High amount of daily screen time was linked with the presence of DE. A strong association between nonsmokers and DE was found, most likely correlated with the nicotine effect of lowering corneal sensitivity, thus causing fewer symptoms. We recommend similar future studies on other medical universities in Romania to support the data provided. A high prevalence of dry eye disease among medical students may indicate a broader public health issue. This information can be used to inform healthcare policies, establish occupational health guidelines, and implement preventive measures for individuals in similar high-stress academic or professional environments. Our aim is to raise awareness of the impact of DE on medical students and its consequences on quality of life. Most risk factors associated with dry eyes are controllable and should represent the first step in starting therapy.

### Electronic supplementary material

Below is the link to the electronic supplementary material.

**Supplementary Material 1**: Questionnaire applied through Google Forms

**Supplementary Material 2**: Romanian translation of OSDI Questionnaire provided by Allergan

## Data Availability

The datasets used and/or analyzed during the current study are available from the corresponding author upon reasonable request.
