# Supplementary material for: Assessment of the prevalence and risk factors for dry eye symptoms among Romanian medical students using the ocular surface disease index – a cross-sectional study
Source: BMC Ophthalmol. 2024 Jan 5;24:12. doi: 10.1186/s12886-023-03260-1 (PMC10768241; doi:10.1186/s12886-023-03260-1)
Supplement: Supplementary file 1 — Supplementary Material 1: Questionnaire applied through Google Forms [file 12886_2023_3260_MOESM1_ESM.docx]

**Supplementary material 1.** Questionnaire applied through Google Forms

1. Sex ;
2. Age ;
3. Year of study;
4. How much time do you spend in front of blue screens every day (including mobile phone, laptops, tablets, television, etc): 1-3 h/ day, 3-5 h/day, 5-8 h/day, >8h/ day;
5. Do you have one of the following risk factors: smoking, contact lens wearer, history of refractive surgery, history of keratoconus, treatment with oral contraceptives, autoimmune disease, atopy, vitamin A deficiency, Congenital cataract, Antimuscarinic treatment, Isotretinoin treatment, No risk factor;
6. OSDI questionnaire (Supplementary material 3.);
