# Supplementary material for: Assessment of the prevalence and risk factors for dry eye symptoms among Romanian medical students using the ocular surface disease index – a cross-sectional study
Source: BMC Ophthalmol. 2024 Jan 5;24:12. doi: 10.1186/s12886-023-03260-1 (PMC10768241; doi:10.1186/s12886-023-03260-1)
Supplement: Supplementary file 2 — Supplementary Material 2: Romanian translation of OSDI Questionnaire provided by Allergan [file 12886_2023_3260_MOESM2_ESM.docx]

**Supplementary Material 2.** Romanian translation of OSDI Questionnaire provided by Allergan

**INDEXUL BOLILOR SUPRAFEȚEI OCULARE** (OSDI ©)

**Vă rugăm să răspundeți la următoarele întrebări bifând căsuța care corespunde cel mai bine răspunsului dvs.**

Ați simțit vreuna din următoarele senzații **în ultima săptămână**:

|  |  | Tot timpul | Majoritatea timpului | Jumătate din timp | Uneori | Niciodată |
| --- | --- | --- | --- | --- | --- | --- |
| 1 | Ochi sensibili la lumină? |  |  |  |  |  |
| 2 | Senzație de nisip în ochi? |  |  |  |  |  |
| 3 | Ochi dureroși? |  |  |  |  |  |
| 4 | Vedere încețoșată? |  |  |  |  |  |
| 5 | Vedere slabă? |  |  |  |  |  |

Problemele cu ochii v-au limitat în efectuarea vreuneia din următoarele activități **în ultima săptămână**:

|  |  | Tot timpul | Majorita-tea timpului | Jumătate din timp | Uneori | Nicio-dată | Nu este cazul |
| --- | --- | --- | --- | --- | --- | --- | --- |
| 6 | Citit? |  |  |  |  |  |  |
| 7 | Șofat pe timp de noapte? |  |  |  |  |  |  |
| 8 | Lucrul la calculator sau la un bancomat (ATM)? |  |  |  |  |  |  |
| 9 | Privitul la televizor? |  |  |  |  |  |  |

Ați simțit disconfort la ochi în vreuna din următoarele situații **în ultima săptămână**:

|  |  | Tot timpul | Majorita-tea timpului | Jumătate din timp | Uneori | Nicio-dată | Nu este cazul |
| --- | --- | --- | --- | --- | --- | --- | --- |
| 10 | Condiții de vânt? |  |  |  |  |  |  |
| 11 | Locuri sau zone cu umiditate redusă (cu atmosferă foarte uscată)? |  |  |  |  |  |  |
| 12 | Spații cu aer condiționat? |  |  |  |  |  |  |

Copyright © 1995 Allergan, Inc.
